# Clinical Relevance of Methylenetetrahydrofolate Reductase Genetic Testing in Autism: A Case Report of Successful Clinical Outcome

**DOI:** 10.7759/cureus.12586

**Published:** 2021-01-09

**Authors:** Fadila ., Praveen Suman, Praveen Kumar, Md Faraz Omair

**Affiliations:** 1 Institute of Child Health, Sir Ganga Ram Hospital, New Delhi, IND; 2 Child Development Clinic, Institute of Child Health, Sir Ganga Ram Hospital, New Delhi, IND; 3 Division of Pediatric Neurology, Institute of Child Health, Sir Ganga Ram Hospital, New Delhi, IND; 4 Department of General Medicine, GreenLife Hospital, Patna, IND

**Keywords:** autism, folic acid supplementation, methylenetetrahydrofolate reductase, autism spectrum disorder

## Abstract

Autism spectrum disorder is an emerging public health issue. The core features of autism spectrum disorder are persistent impairment in reciprocal social communication and interaction and restricted, repetitive patterns of behavior or interests. We now know that it encompasses disorders previously referred to as early infantile autism, childhood autism, Kanner autism, high-functioning autism, atypical autism, Asperger disorder, childhood disintegrative disorder, and pervasive developmental disorder not otherwise specified. While it is agreed that the etiology of autism spectrum disorder is largely unknown, certain environmental and genetic factors may be responsible for the disease. In particular, emerging evidence has suggested the role of C677T polymorphism in the methylenetetrahydrofolate reductase (MTHFR) gene as a possible risk factor. We present the case of a two-year-old boy with high risk for autism who was found on advanced investigation to have heterozygous polymorphism for MTHFR. This prompted us to add folic acid to his therapeutic regime. He was treated with high-dose folic acid along with conventional intervention, and went on to make excellent recovery. We conclude that pharmacological intervention has the potential to improve outcome in a subgroup of autistic children.

## Introduction

Autism is classified as a subgroup of autism spectrum disorders. Autism spectrum disorder is a heterogeneous group of neurodevelopmental disorders affecting communication and behavior whose symptoms become apparent during the early developmental years of life, most commonly by three years of age [1]. There has been an increase in the reported cases of autism spectrum disorders which can be attributed to better diagnostic tools and increased awareness among both physicians and parents. It is generally accepted that autism spectrum disorders have multifactorial inheritance with 90% genetic background [2]. Certain genetic disorders such as Fragile X syndrome and Rett syndrome also have autistic features, and these conditions must be excluded wherever relevant during the clinical evaluation of children. In children without underlying known causes of autism, a particular area of interest has developed in another set of genes, which could have massive therapeutic potential. Recent studies have revealed that genes involved in the folate/homocysteine pathway may be risk factors and/or responsible for features seen in autistic children. Genetic variants affect each patient differently. Some patients remain asymptomatic, while others with the same aberration in genetic material might have serious, long-term health problems. This heterogeneity involves both locus and allelic heterogeneity. Methylenetetrahydrofolate reductase (MTHFR) is one of the most important enzymes in the folate pathway. C677T polymorphism in the MTHFR gene is associated with a decrease in enzymatic activity to 35-70% in homozygotes. Several studies have shown that heterozygous genotype for the MTHFR C677T exhibits more behavioral problems and/or more severe problematic behaviors than homozygous individuals. A recent meta-analysis has also supported significant associations between autism spectrum disorders and MTHFR polymorphism [3]. We report a patient with heterozygous MTHFR genetic polymorphism treated with high-dose folic acid that resulted in improved neurological and developmental outcomes.

## Case presentation

Our patient was a two-year-old child who was first in birth order, born eight weeks premature, with a very low birth weight, to a primigravida mother of 23 years of age. In view of prematurity and respiratory distress shortly after birth, he was managed as a case of respiratory distress syndrome with surfactant administered on days one and two of life. He was also treated for neonatal hyperbilirubinemia on day three of life for 24 hours with phototherapy alone. No exchange transfusion was required. He was discharged home on day eight after establishing oral feeds and on room air. Discharge neonatal check was normal. He passed the neonatal hearing test, and since birth, his medical history had been unremarkable for significant medical concerns. His parents had a nonconsanguineous marriage, and there was no family history of any significant childhood illness, including speech delays and spontaneous abortions. He was noticed to have abnormal behavior by his mother at one year of age. There were also developmental concerns expressed by the mother. The child did not interact well with his parents and preferred to express his needs with physical gestures rather than verbally, but he could speak bisyllables when coaxed.

Upon evaluation by a local pediatrician, his thyroid stimulating hormone level (TSH) was found to be raised at 18 months of age, for which he was started on levothyroxine. The abnormal behavior persisted. By now, he was not using any words but made growling noises when upset and throw things around. He was not making eye contact, choosing to look toward the ground or at the ceiling when being talked to. In other domains of development, he was keeping up with his peers, and no concerns were expressed. Age-appropriate vision testing was carried out which was normal. Upon evaluation at our child development center, he was labeled as high risk of autism as per the Modified Checklist for Autism in Toddlers scoring system at two years of age. His TSH levels had normalized by now. We liaisoned with the neurology team to rule out organic causes for his behavior and development. Blood tests including lead levels, full blood count, TSH, and electrolytes were normal. He tested positive for MTHFR mutation (heterozygous), factor V Leiden mutation (heterozygous), and plasminogen activator inhibitor type 1 deficiency (homozygous). These findings led us to start him on high-dose folic acid (2.5 mg, once a day, orally, daily) along with behavioral training and speech therapy at our center. On follow-up three months later, the mother reported improved behavior with the child using words to express his needs, albeit infrequently. He was now making some eye contact when addressed directly. When Childhood Autism Rating Scale (CARS) was performed six months later at 30 months of age, it was not indicative of any feature of autism. No adverse effects of drug therapy were reported. Regular follow-up examinations have reported improvements in general behavior and speech. At four years of age, he was able to interact well with children in his playschool, loved to share lunch and toys, and recite stories.

## Discussion

Normal activity of MTHFR is required for normal genome methylation and imprinting. The main neuroanatomical abnormalities in autistic children at birth are mainly in the hippocampus and prefrontal cortex regions. Cerebral folate levels are reduced in such children, and supplementation with high-dose folic acid improves the folate level, particularly in those with MTHFR mutation. Maternal folic acid deficiency during pregnancy is also an established risk factor for having children with autism spectrum disorders [4]. Numerous studies have reported that dysfunctional folate-methionine pathway enzymes may play an important role in the pathophysiology of autism [5]. In such children, a three-month folic acid supplementation has been shown to improve autistic features, particularly cognition and communication [6], as was the case in our patient. We undertook rapid evaluation, with early intervention, and evaluated our patient’s response using CARS, which is a 15-point behavioral rating scale developed to both identify autism as well as define the severity of the disorder [7]. The child continued on folic acid supplementation, though the dose was reduced once improvement was noticed. Currently, he is on 2.5 mg oral tablets three days a week. There have been several case reports highlighting the therapeutic potential of folic acid in autistic children. A recent placebo randomized control trial reported from the United States also highlighted significant improvements, evaluated using the Autism Diagnostic Observation Schedule score after 12 weeks of use [8]. However, to the best of our knowledge, to date, there is a lack of consensus data on the dose and duration of treatment to be offered. Some case reports also suggest the use of a casein-free, gluten-free diet in children with autism spectrum disorder. There is also no dearth of fad diets and sham supplements claiming to “cure” autism. We want to emphasize that the role of behavioral and educational therapy cannot be discounted at this stage. We need large multicentric studies to enable guidelines so that scores of children living with autism spectrum disorder could benefit. However, it is worth noticing that a simple folic acid supplementation could significantly improve autism spectrum disorders.

## Conclusions

Genetic workup for MTHFR gene polymorphism must be considered in children diagnosed with autism spectrum disorder, particularly when basic workup has been noncontributory. Early intervention using high-dose folic acid in children with high risk of autism who are heterozygous for MTHFR mutation favors a better response compared to those who are not supplemented but receive the standard behavior and speech therapy at dedicated centers. Pharmacologic intervention may increase the ability of children with autism spectrum disorders to benefit from educational and other interventions.
